# Detection of House Dust Mite-derived DNA in Human Lung Tumors by Whole-Genome Sequencing

**DOI:** 10.21203/rs.3.rs-10438367/v1

**Published:** 2026-08-27

**Authors:** Sunandini Sharma, Tongwu Zhang, John McElderry, Olivia Lee, Phuc Hoang, Liping Zeng, Nicholas Webster, Ludmil Alexandrov, Eyal Raz, Maria Teresa Landi, Samuel Bertin

**Affiliations:** University of California San Diego; National Cancer Institute; National Cancer Institute; National Cancer Institute; National Cancer Institute; University of California San Diego; University of California San Diego; University of California San Diego; University of California San Diego; National Cancer Institute; University of California San Diego

**Keywords:** Lung cancer, Whole-genome sequencing, House dust mites, Environmental exposure, Tumor microenvironment, Microbiome

## Abstract

Lung cancer in never-smokers (LCINS) accounts for an increasing proportion of lung cancer cases, yet its risk factors remain poorly understood. House dust mites (HDM) are common aeroallergens that induce airway inflammation, but their potential contribution to lung cancer is unknown. We analyzed unmapped whole-genome sequencing reads from 783 lung cancers from the Sherlock-*Lung* (n = 621 never-smokers) and EAGLE (n = 162 smokers) cohorts, including 328 matched adjacent normal lung tissues. After removal of human sequences, reads were aligned to reference genomes from the two major HDM species and confirmed by BLAST. Samples with top BLAST matches were classified as HDM-detected. Associations between HDM detection and genomic, microbiome, and bulk RNA-seq-derived immune features were evaluated. HDM-derived DNA was detected at low abundance in a subset of tumors and adjacent normal tissues, with higher detection frequencies in tumors than matched normal tissues and in smokers than never-smokers. In LCINS tumors, HDM detection was not associated with tumor mutational burden or recurrent driver alterations but was associated with modest differences in immune cell composition and a limited but reproducible bacterial co-detection pattern. These findings provide a foundation for investigating aeroallergen-derived DNA signatures and their potential relationship to the lung tumor microenvironment.

## Introduction

Lung cancer is responsible for approximately 1.8 million deaths per year and remains the leading cause of cancer-related death worldwide^1^. Tobacco smoking is the major risk factor for lung cancer; however, up to 25% of cases are diagnosed in never-smokers^2,3^. Both smokers and never-smokers are mainly affected by non-small cell lung cancer (NSCLC), predominantly lung adenocarcinoma (LUAD)^4^, but LUAD arising in smokers and never-smokers has different molecular profiles and distinct tumor microenvironments (TME)^2,3^, which affect response to treatments, including novel targeted therapies and immunotherapies^2,3,5^. As smoking rates have declined, the proportion of lung cancer in never-smokers (LCINS) has been increasing^2,3^. LCINS is now the seventh leading cause of cancer deaths in the US and the fifth leading cause worldwide^2^. LCINS is associated with other risk factors such as exposure to radon gas, air pollution, and genetic factors^2,3^, as well as pre-existing chronic inflammatory lung diseases such as chronic obstructive pulmonary disease (COPD)^6,7^ and asthma^8,9^. However, most LCINS cases have no known risk factors, and the environmental exposures and biological mechanisms that increase lung cancer risk in never-smokers remain largely unknown. Accordingly, a better understanding of environmental factors that can trigger tumor-promoting inflammation in the lungs^10^ may help clarify the etiology of LCINS.

House dust mites (HDM) are a major cause of perennial allergic rhinitis, atopic dermatitis, and asthma, affecting approximately 1–2% of the global population, representing an estimated 65–130 million individuals worldwide^11,12^. The most common mite species are *Dermatophagoides pteronyssinus* (European HDM) and *Dermatophagoides farinae* (American HDM), which are widely distributed across temperate and tropical regions and represent the primary sources of clinically relevant HDM allergens worldwide^11,12^. HDM are well recognized air pollutants and inhalant allergens that disrupt the airway epithelial barrier, immune homeostasis, and trigger inflammation^12,13^. In addition, HDM exposure has been reported to induce oxidative damage and DNA double-strand breaks in airway epithelial cells, leading to increased pro-inflammatory cytokine production and epithelial apoptosis^14,15^, and potentially contributing to mutagenic processes associated with lung tumorigenesis.

Taken together, these observations suggest that the consequences of chronic HDM exposure may extend beyond allergic disease to include biological mechanisms implicated in lung carcinogenesis. Consistent with this hypothesis, our recent studies have shown that chronic HDM exposure promotes sustained lung inflammation, immune-suppressive microenvironment remodeling, and accelerated lung cancer progression in multiple mouse models^16,17^. However, no studies to date have directly evaluated the potential impact of HDM exposure on lung cancer development or progression in humans. Given that microbial DNA derived from bacteria, viruses, and fungi has previously been detected in lung tumors^18,19,20^, albeit at low biomass^21^, and may contribute to tumor-associated biological processes, we hypothesized that HDM-derived DNA fragments may also be present within the lung tumor microenvironment and influence tumor biology. To investigate this possibility, we leveraged genomic and transcriptomic datasets from the Sherlock-*Lung* study, which was designed to investigate LCINS etiology in treatment-naïve patients^22^, together with data from smokers enrolled in the EAGLE study^23^. Because HDM reference genomes are currently available at different assembly levels, we first performed a comparative genomic characterization of these resources before assessing whether HDM-derived DNA is detectable in lung tumors and examining its associations with clinical, genomic, immune, and microbial features.

## Methods

### Ethics statement

The National Cancer Institute (NCI) exclusively received de-identified human tissue specimens and associated data from collaborating centers, had no direct interaction with study participants, and did not use or generate identifiable private information. Therefore, this study was classified as “Not Human Subjects Research (NHSR)” under the U.S. Federal Common Rule (45 CFR 46.102(e)). Human tissue specimens analyzed in this study were obtained from two previously established cohorts: the Sherlock-Lung study and the Environment and Genetics in Lung Cancer Etiology (EAGLE) study. Sherlock-Lung specimens were obtained from collaborating biobanks, including the IUCPQ Tissue Bank, site of the Quebec Respiratory Health Network Biobank and the Fonds de recherche du Québec–Santé (FRQS) (www.tissuebank.ca), and the INCLIVA Biobank (PT17/0015/0049), integrated within the Spanish National Biobanks Network and the Valencian Biobanking Network. These specimens were collected and processed under protocols approved by their respective Institutional Review Boards (IRBs) or Ethics and Scientific Committees. EAGLE participants provided written informed consent before enrollment, and the study was approved by the IRBs of each participating hospital and university in Italy and by the NCI, Bethesda, Maryland. All methods were carried out in accordance with relevant guidelines and regulations. Written informed consent was obtained from all participants prior to enrollment in their respective studies.

### Lung cancer cohort and sequencing data

Whole-genome sequencing (WGS) and bulk RNA-seq data were obtained from the Sherlock-Lung^21,22^ and EAGLE^23^ studies. An initial set of 1,153 WGS tumor samples was evaluated. To minimize platform-related technical variability and ensure consistent sequencing characteristics, publicly available WGS samples lacking verified sequencing platform information (n = 153) and HiSeq X-sequenced samples (n = 217) were excluded, resulting in a final cohort of 783 NovaSeq 6000-sequenced lung tumors. Matched adjacent normal lung WGS data were available for 328 subjects and were used for paired tumor-normal analyses. Bulk RNA-seq data were available from 627 of the 783 tumors and were used for immune deconvolution analyses. Genetic ancestry was inferred from WGS data. Clinical, epidemiological, and genomic annotations, including smoking status, histological subtype, tumor stage, age at diagnosis, sex, genetic ancestry, asthma and chronic obstructive pulmonary disease (COPD) history, tumor mutational burden (TMB), study site, and survival information, were integrated for downstream analyses (**Supplementary Table 1**).

### HDM reference genomes

Genome assemblies for *Dermatophagoides farinae* (American HDM) and *Dermatophagoides pteronyssinus* (European HDM) were obtained from publicly available NCBI databases and are summarized in Table 1. Because assembly completeness varied across available resources, we used multiple reference builds spanning chromosome-, scaffold-, and contig-level assemblies.

### Host read removal and alignment

Raw sequencing reads underwent quality control followed by removal of human-derived reads through alignment to the T2T-CHM13 human reference genome, as previously described^23^. Reads that did not align to the human reference were retained and separated into paired-end and singleton reads for downstream analysis. Non-human reads were aligned to *Dermatophagoides* reference genomes using bwa-mem2 (version: 2.2.1) with default parameters and multithreading. Alignments were performed independently for each assembly (chromosome-, scaffold-, and contig-level) to enable comparative analyses across reference types. Resulting SAM files were converted to BAM format using samtools (version: 1.23), followed by coordinate sorting.

### Post-alignment filtering

Mapped reads were extracted by excluding unmapped alignments (SAM flag -F 4). To ensure high-confidence alignments, reads were further filtered based on mapping quality (MAPQ ≥ 30). In addition, aligned segments were parsed from CIGAR strings, and only reads containing ≥ 40 bp of matched bases were retained. Filtered reads were exported in FASTA format for downstream taxonomic validation and analysis. These filters were designed to minimize low-confidence and multi-mapping reads.

### Taxonomic validation using BLAST

To confirm taxonomic specificity, candidate HDM-p and post-filtered reads were queried against the NCBI nucleotide (nt) database using BLASTn. Searches were performed in parallel using GNU Parallel, with each query processed using two threads (-num_threads 2). Results were reported in tabular format (-outfmt 6) including alignment statistics, sequence identity, alignment length, and taxonomic annotations (including taxonomic IDs and scientific names). For each read, the top-scoring BLAST hit was used for taxonomic assignment. Reads with top hits corresponding to *Dermatophagoides* species were considered HDM-supported. The number of BLAST-supported HDM reads was quantified per sample for each reference assembly. Within each sample, BLAST hits were first sorted by query sequence identifier (qseqid) and bitscore in descending order so that the highest-scoring alignments for each read were prioritized. For each query read, the maximum bitscore was identified, and taxonomic assignment was based only on hits tied at that top bitscore. HDM signal detection was performed using a permissive exploratory approach based on alignment of unmapped reads (≥ 1 supporting read). A read was considered supported for *Dermatophagoides farinae* only when at least one of its top-scoring BLAST hits had a subject title (stitle) matching *D. farinae*. If multiple hits shared the same highest bitscore, the read was still counted as HDM-supported provided that one of those equally scoring best hits matched the target species. This strategy was used to avoid overcounting lower-scoring secondary matches and to ensure that species assignment reflected the best-supported BLAST evidence for each query. Each query read was counted only once, regardless of the number of alignments returned by BLAST. The final output for each sample was the number of unique reads whose best BLAST-supported assignment corresponded to *D. farinae.* The same analysis was applied to the European HDM. This analysis was run for both paired-end and singleton reads to identify high confidence HDM-supported reads in tumor and matched normal samples. Given the low abundance of microbial reads in our lung cancer WGS dataset, this approach was used to maximize sensitivity for hypothesis generation.

### HDM detection classification

Samples were classified based on the presence or absence of BLAST-supported HDM reads. Because BLAST-supported read counts were generally low, samples containing at least one BLAST-supported HDM read were classified as HDM-detected, whereas samples without any BLAST-supported HDM reads were classified as HDM-non-detected. Classification was performed independently for each HDM reference assembly and for tumor and matched normal samples.

### Coverage analysis

Genome-wide coverage profiles were generated by calculating cohort-level median depth across fixed genomic bins for each HDM assembly. Coverage was assessed before and after filtering to evaluate the impact of alignment quality and filtering criteria. Genome-wide coverage of HDM-aligned reads was assessed using Mosdepth. For each sample, indexed BAM files containing filtered, mapped reads were used as input. Coverage was calculated across the reference genome in fixed-width bins of 10 kb (--by 10000) to obtain a uniform representation of read distribution. Mosdepth was run with the -n flag to suppress per-base output and focus on region-level coverage estimates. Resulting coverage files provided per-bin depth values, which were used to generate genome-wide coverage profiles for each HDM assembly. Coverage distributions were summarized at the cohort level by computing median depth across bins, enabling comparison of coverage patterns across samples and assemblies. These profiles were used to evaluate the distribution, sparsity, and localization of HDM-aligned signal across the genome. Genome-wide coverage profiles were aggregated across samples using fixed-width binning derived from mosdepth output. For each sample, per-bin depth values (10 kb resolution) were extracted from mosdepth.regions.bed files. Coverage data from all samples were combined into a unified dataset, and for each genomic bin (defined by chromosome, start, and end coordinates), the median depth across the cohort was computed to obtain a robust summary of coverage. To enable visualization of genome-wide coverage patterns, chromosomes were ordered and concatenated into a continuous coordinate system. For each bin, the midpoint position was calculated and offset by cumulative chromosome lengths to generate a genome-wide coordinate. The resulting profiles represent cohort-level median depth across the HDM reference genome and were used to assess the distribution and localization of HDM-aligned signal. This analysis was performed similarly for all other assemblies.

### Overlap analysis across assemblies

Overlap of HDM detection calls across assemblies was evaluated using intersection-based approaches. Detection patterns were compared across chromosome-, scaffold-, and contig-level assemblies for both tumor and normal samples.

### Functional annotation of HDM-supported reads

BLAST-supported HDM reads were functionally annotated based on the gene descriptions of their top-scoring BLAST hits. Reads were subsequently grouped into functional categories, including structural components, metabolic processes, proteases/peptidases, and uncharacterized loci, using keyword-based classification of the corresponding HDM annotations.

### TMB calculations

TMB was calculated from WGS data as previously described^24^ based on the total number of filtered and annotated coding and noncoding variants. Coding variants included nonsynonymous, frameshifts, and splicing variants, whereas all variant types from ANNOVAR-annotated variants were classified as noncoding. TMB was normalized to mutations per megabase by dividing the total number of variants by the size of the human reference genome (GRCh38; approximately 3,000 Mb).

### Genomic association analyses

Associations between HDM detection and recurrent genomic alterations were evaluated using multivariable logistic regression models performed separately in never-smokers and smokers. Models were adjusted for clinical stage, study site, continent, and genetic ancestry. Associations are reported as adjusted odds ratios (ORs) with 95% confidence intervals (CIs). Differences in TMB between HDM-detected and HDM-non-detected tumors were assessed using the Wilcoxon rank-sum test.

### Immune deconvolution analysis

Immune and stromal cell-type abundances were inferred from bulk RNA-seq data using the xCell algorithm^25^, which has been shown to provide robust estimates of cell-type composition^26^. Differences in inferred cell-type abundances between HDM-detected and HDM-non-detected tumors were evaluated using two-sided Welch’s t-tests. Figures display nominal (uncorrected) p-values for visualization, whereas statistical significance was determined after FDR correction.

### Microbiome association analysis

Microbiome association analyses were performed using previously generated contamination-filtered microbiome profiles from the Sherlock-Lung study^21^. A total of 617 never-smoker tumors were included after exclusion of four tumors during contaminant filtering in the original study. Microbiome data were not available for the EAGLE cohort; therefore, smokers were not included in these analyses. Previously generated relative abundance estimates for 495 bacterial and 5 fungal genera were compared between HDM-detected and HDM-non-detected tumors. Genus-level differential abundance analyses were performed separately for each HDM reference assembly and resulting p-values were adjusted for multiple testing using the Benjamini–Hochberg FDR method.

### Statistical analysis

All statistical analyses were performed using R (v4.5.1) and Python (v3.11). Continuous variables were compared using two-sided Welch’s t-tests unless otherwise specified. Multiple testing corrections were applied using the Benjamini-Hochberg FDR method where applicable. A two-sided p-value < 0.05 (FDR < 0.05 for multiple comparisons) was considered statistically significant.

### Graphical illustrations

Graphical illustrations were created using BioRender. Figures were designed and exported in accordance with the platform’s licensing and publication guidelines.

## Results

### Study design, cohort selection, and analysis workflow

To investigate the presence of HDM-derived DNA in lung tumors, we obtained unmapped non-human reads from a previously published analysis of the Sherlock-Lung and EAGLE whole-genome sequencing (WGS) datasets^21^ and used them as the starting point for our analysis. Because currently available HDM reference genomes are represented by chromosome-, scaffold-, and contig-level assemblies, we first performed a comparative genomic characterization of these resources to guide subsequent alignment-based detection of HDM-derived DNA in human WGS datasets.

The initial dataset comprised 1,153 WGS tumor samples, including HiSeq X-sequenced samples (n = 217), NovaSeq 6000-sequenced samples (n = 783), and publicly available WGS samples for which the sequencing could not be verified (n = 153). Total read counts differed significantly across these groups, with NovaSeq 6000 samples showing the highest median sequencing depth (2.36 × 10^9^ reads), followed by HiSeq X samples (2.13 × 10^9^ reads) and publicly downloaded samples (1.82 × 10^9^ reads) (**Supplementary Fig. 1A**). Tumor samples were obtained from multiple Sherlock-Lung study sites, whereas EAGLE samples originated from a single study (**Supplementary Fig. 1B**).

To minimize confounding by sequencing platform, publicly downloaded samples lacking verified sequencing platform information (n = 153) were excluded from further analysis. The remaining unmapped non-human reads (paired-end and singletons) were aligned to the selected HDM reference genomes, and HDM abundance was quantified as reads per million total reads (RPM; HDM-aligned reads/total reads × 10^6^). Principal component analysis (PCA) of HDM RPM values revealed clear clustering by sequencing platform (**Supplementary Fig. 1C**). Consistent with this observation, HDM-aligned RPM values were substantially higher in NovaSeq 6000 samples than in HiSeq X samples across all HDM reference assemblies (**Supplementary Fig. 1D**).

To reduce nonspecific alignments from short sequences, HDM-aligned reads were further filtered to retain only high-quality alignments with a mapping quality score (MAPQ) ≥ 30 and a matched alignment length (CIGAR-matched length) of at least 40 bp. Following filtering, HiSeq X samples retained very few high-confidence HDM-aligned reads, whereas NovaSeq 6000 samples retained substantially more reads meeting these stringent criteria (**Supplementary Fig. 1E and F).** The limited post-filtering signal in HiSeq X samples likely reflected technical differences, including sequencing chemistry, library preparation, read depth, read quality, or cross-mapping artifacts, rather than biological variation. Consequently, downstream analyses were restricted to NovaSeq 6000-sequenced samples (n = 783), which represented the largest and most technically consistent sequencing platform group. Batch correction was not applied because the pronounced platform effect was unlikely to represent a simple additive or multiplicative batch effect. Instead, correcting for platform differences could have introduced artifacts or artificial bimodality in HDM read distributions.

**Figure 1:**
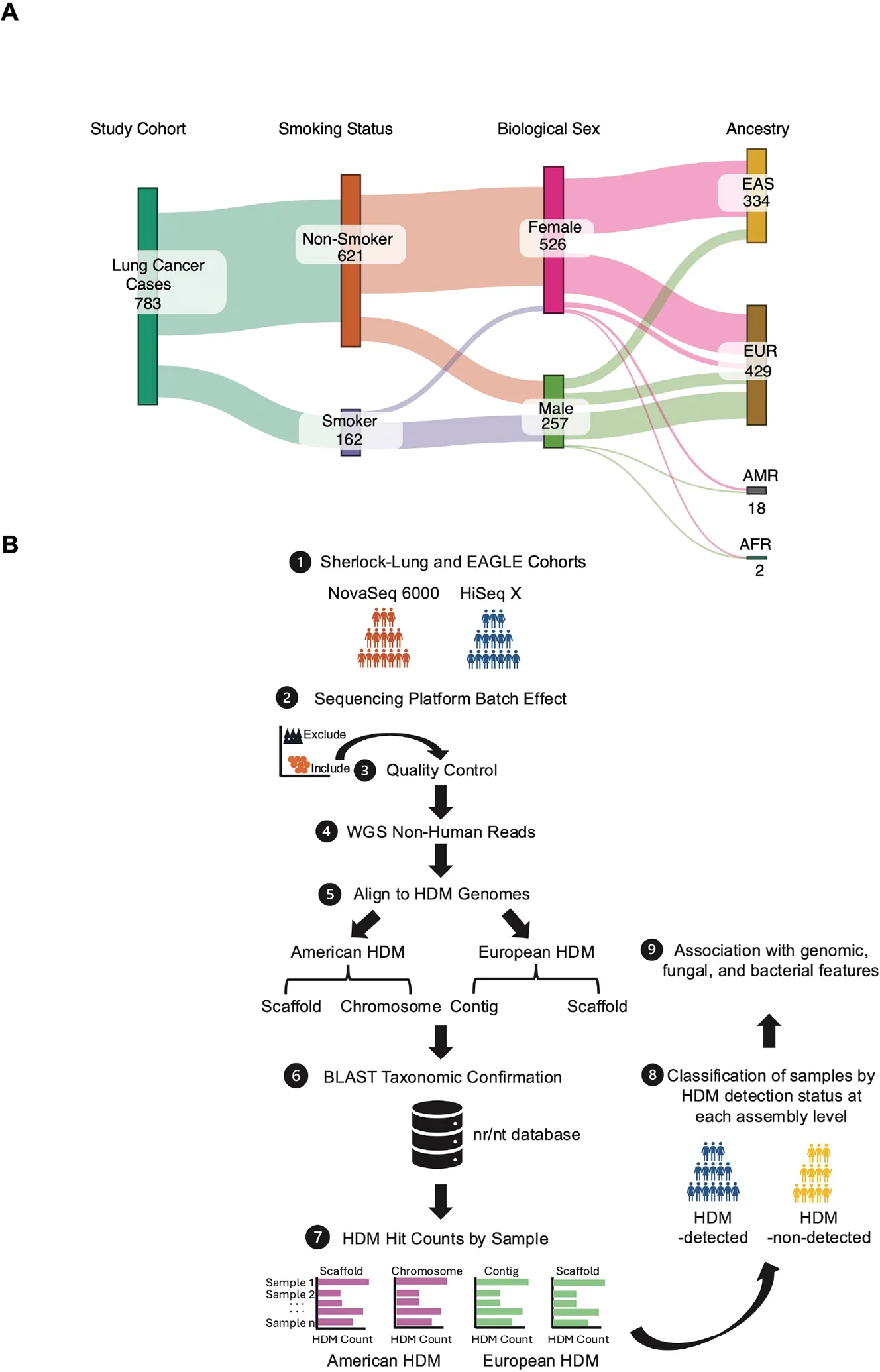
Study cohort characteristics and workflow for HDM-derived DNA detection. A) Sankey plot summarizing the clinical and demographic characteristics of the final lung cancer cohort (n = 783). Patients are grouped by smoking status, biological sex, and WGS-derived genetic ancestry (European [EUR], East Asian [EAS], Admixed American [AMR, and African [AFR]). Node and link colors distinguish categories, and link widths are proportional to the number of subjects represented. B) Schematic overview of the computational workflow used to detect HDM-derived DNA in lung WGS data. Following assessment of sequencing-platform batch effects and quality control, unmapped non-human reads were aligned to chromosome-, scaffold-, and contig-level reference assemblies of the American and European HDM genomes. Candidate HDM-aligned reads were validated by BLAST against the NCBI nr/nt database, and BLAST-supported reads were summarized for each sample to classify lung tumors and matched adjacent normal lung tissues as HDM-detected or HDM-non-detected. These classifications were subsequently used for downstream association analyses of clinical, genomic, immune, and microbiome features.

The final study cohort consisted of 783 subjects with available clinical annotations (**Supplementary Table 1**), of whom 328 had matched tumor-adjacent normal lung WGS data. Bulk RNA-seq data were available from 627 of the 783 tumors and were used for immune deconvolution analyses. Among the 783 patients, 621 were never-smokers and 162 were smokers. The cohort comprised 526 females and 257 males and was predominantly of European (EUR; n = 429) or East Asian (EAS; n = 334) ancestry, with a small number of patients of Admixed American (AMR; n = 18) or African (AFR; n = 2) ancestry. (Fig. 1A). The median age at diagnosis was 64.3 years. LUAD was the predominant histological subtype, accounting for 703 of 783 tumors (89.8%). The remaining 80 tumors (10.2%) comprised non-LUAD histologies, including lung squamous cell carcinoma (LUSC), carcinoid tumors, large cell carcinoma, and other rare histological subtypes; 78 occurred in never-smokers and 2 in smokers. Clinical stage at diagnosis was Stage I (n = 451), Stage II (n = 134), Stage III (n = 131), Stage IV (n = 39), or unknown (n = 28). A history of asthma was uncommon, being reported in only 13 patients (1.7%; 11 never-smokers and 2 smokers), whereas 571 patients (72.9%) had no history of asthma and asthma status was unavailable for 199 patients (25.4%) (**Supplementary Table 1**).

To sensitively detect HDM-derived DNA across this heterogeneous WGS cohort, we developed a stringent analytical framework combining host-depletion, comparative evaluation of HDM reference genomes, multi-reference alignment and BLAST-based validation (Fig. 1B). Residual human sequences were removed by alignment to the T2T-CHM13 human reference genome, and the remaining non-human reads were aligned to chromosome-, scaffold-, and contig-level reference assemblies of the American and European HDM genomes. Candidate HDM-aligned reads were subsequently validated by BLAST against the NCBI nr/nt database. Samples containing BLAST-supported HDM reads were classified as HDM-detected or HDM-non-detected and used for downstream association analyses of clinical, genomic, immune, and microbiome features.

### HDM reference genome selection and species assembly-level comparison

To establish a robust framework for HDM detection, we first compared the currently available reference genome assemblies for *Dermatophagoides pteronyssinus* (European HDM) and *Dermatophagoides farina* (American HDM). As of late 2025, NCBI hosted three scaffold-level and one contig-level assembly for European HDM (NCBI Taxonomy ID: 6956), whereas American HDM (NCBI Taxonomy ID: 6954) was available at chromosome and scaffold assembly levels. Because chromosome-level assemblies were unavailable for European HDM, scaffold-level assemblies for both species were selected for the primary cross-species comparison to ensure comparable assembly resolution. The American chromosome-level assembly and the European contig-level assembly were included in secondary analyses to evaluate whether HDM detection and downstream association were influenced by assembly resolution.

**Figure 2:**
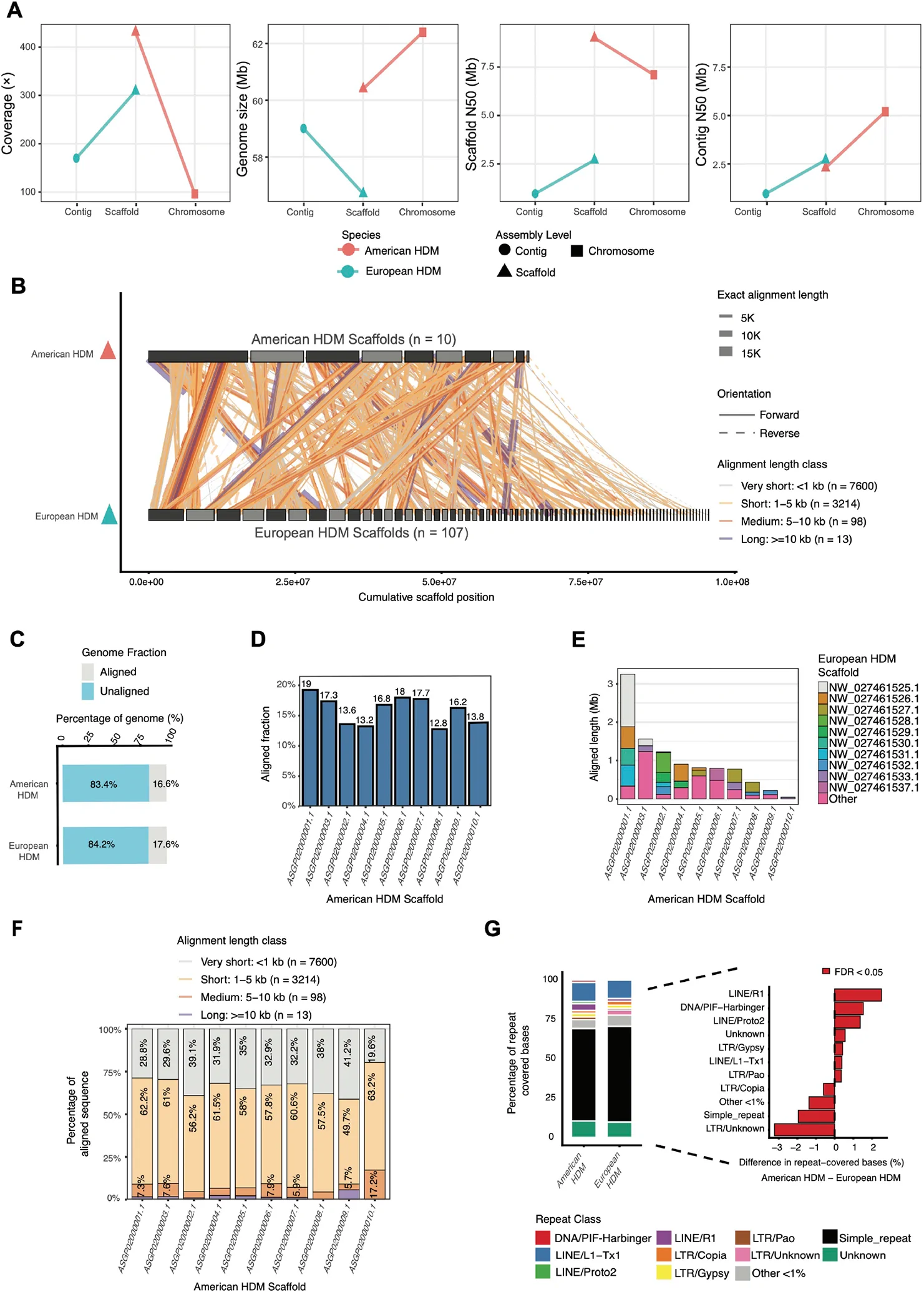
Comparative genome assembly features, synteny, aligned genomic regions, and repeat composition of American and European HDM genomes. **A)** Comparison of assembly characteristics for American and European HDM reference genomes, including genome coverage, genome size, scaffold N50, and contig N50 across contig-, scaffold-, and chromosome-level assemblies. **B)** Whole genome synteny plot comparing the scaffold-level assemblies of American HDM (n = 10 scaffolds) and European HDM (n = 107 scaffolds). Links represent aligned blocks between the two assemblies and are colored according to alignment-length category. Both forward and reverse alignments are shown. **C)** Genome-wide aligned and unaligned fractions between American and European HDM scaffold assemblies. Approximately 16.6% of the American HDM genome and 17.6% of the European HDM genome aligned between species. **D)** Fraction of each American HDM scaffold aligned to the European HDM scaffold assembly. **E)** Distribution of aligned sequence lengths between American HDM scaffolds and individual European HDM scaffolds. The “Other” category represents alignments to additional smaller European HDM scaffolds not shown individually. **F)** Distribution of syntenic alignment-length classes between American and European HDM scaffold assemblies. Stacked bars show the proportion of aligned sequence within each alignment-length category for individual American HDM scaffolds. **G)** Repeat composition of aligned genomic regions between American and European HDM assemblies. The left panel shows the proportion of repeat-covered bases assigned to each repeat class in each species. The right panel shows differences in repeat-class abundance between species, with positive values indicating enrichment in American HDM and negative values indicating enrichment in European HDM. Red bars denote repeat classes with FDR < 0.05.

The selected reference assemblies differed in genome size, completeness, contiguity, and repeat composition (Table 1 and Fig. 2A). For both HDM species, scaffold-level assemblies exhibited higher genome coverage than alternative assemblies available for the same species. Genome sizes ranged from approximately 56–62 Mb across assemblies, with the American HDM chromosome-level assembly exhibiting the largest genome size, consistent with increased assembly completeness. American HDM assemblies were generally more contiguous than European HDM assemblies, with fewer scaffolds and higher N50 values. For European HDM, contiguity improved from the contig-level to scaffold-level assembly, whereas the American chromosome-level assembly resolved 10 chromosomes and exhibited the highest structural continuity overall. Importantly, these differences largely reflect assembly level and completeness rather than intrinsic biological differences between the two HDM species. Despite these differences, Benchmarking Universal Single-Copy Orthologs (BUSCO) analysis demonstrated comparable completeness between American and European HDM reference genomes, with 91.1% and 91.5% complete BUSCOs, respectively, supporting their suitability for downstream comparative analyses (**Supplementary Table 2**).

For cross-species HDM comparison, scaffold-level assemblies available for both species were used. Whole-genome alignment revealed substantial genomic divergence between American and European HDM. Most alignments consisted of short blocks (< 5 kb), whereas long alignments (≥ 10 kb) were relatively uncommon, indicating that homologous regions are predominantly fragmented rather than organized into large, conserved segments (Fig. 2B). Approximately 16.6% of the American HDM genome and 17.6% of the European HDM genome aligned non-redundantly, with a weighted mean sequence identity of 85.6% across aligned regions (Fig. 2C and **Supplementary Table 3**). MUMmer analysis identified 10,925 alignment blocks between the two genomes. Across the 10 largest American HDM scaffolds, the aligned fraction ranged from 12.8% to 19.0%, with scaffold ASGP02000001.1 exhibiting the highest aligned fraction and ASGP02000008.1 the lowest (Fig. 2D). Despite variation in scaffold size, the proportion of aligned sequence was relatively consistent across scaffolds, indicating that sequence similarity is broadly distributed throughout the American HDM genome. In terms of absolute aligned sequence length, scaffold ASGP02000001.1 contributed the largest amount of aligned sequence (> 3 Mb), whereas the remaining scaffolds showed progressively smaller contributions (Fig. 2E). Analysis of alignment-length distributions showed that 1–5 kb alignment blocks accounted for approximately 50–63% of aligned sequence across most American HDM scaffolds (Fig. 2F). In contrast, long alignments (≥ 10 kb) contributed only a small fraction of aligned sequence, indicating that only a modest fraction of the two genomes shares detectable sequence similarity.

We next compared repeat content between the two HDM species, as differences in repetitive elements may influence alignment behavior and species-specific detection. RepeatMasker annotation revealed that both HDM genomes were enriched for repetitive elements, with simple repeats representing the dominant repeat class in each species (Fig. 2G).

However, the relative abundance of several transposable element families differed between American and European HDM (Fig. 2G, **left panel**). Compared with European HDM, American HDM showed higher relative abundance of LINE/R1, DNA/PIF-Harbinger, LINE/Proto2, Unknown, LTR/Gypsy, LINE/L1-Tx1, and LTR/Pao elements, whereas European HDM showed enrichment of LTR/Unknown, Simple_repeat, and LTR/Copia elements (Fig. 2G, **right panel, and Supplementary Table 4**). These findings indicate that, although both genomes are repeat-rich, they exhibit distinct repeat landscapes.

Together, these analyses demonstrate that American and European HDM reference genomes differ substantially in sequence organization despite broadly similar genome sizes, with only ~ 16–18% non-redundant genome alignment and predominantly short homologous regions. This comparative genomic characterization established the analytical framework for all subsequent HDM detection analyses and supported the use of species-specific reference genomes evaluated across multiple assembly levels.

### Tumor-normal comparison of HDM-aligned reads after stringent mapping filters

Given the relatively fragmented nature of available HDM reference assemblies, we applied stringent alignment filters requiring a matched alignment length of ≥ 40 bp in the CIGAR string, MAPQ ≥ 30, and exclusion of secondary and supplementary alignments. Prior to filtering, coverage tracks showed broad genome-wide alignment signal across HDM assemblies, whereas post-filtering tracks exhibited substantially reduced and more discrete alignment peaks (**Supplementary Fig. 2**). To summarize cohort-wide coverage patterns while minimizing the influence of high-depth outlier regions, the median of mean coverage values across genomic bins was used as the representative signal.

**Figure 3:**
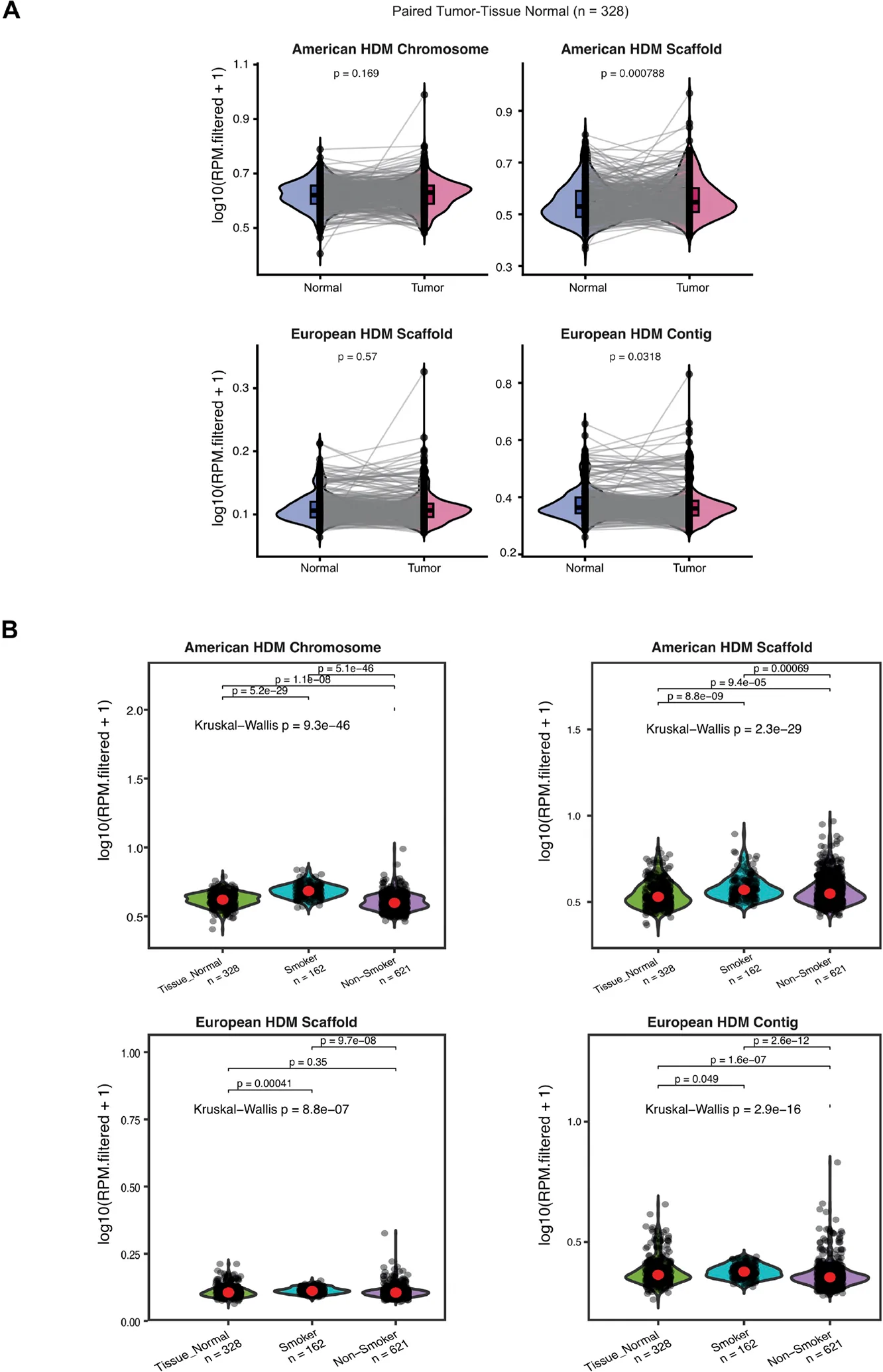
Candidate HDM-aligned read levels across matched sample types and smoking status. **A)** Paired comparison of candidate HDM-aligned read levels between tumor and matched adjacent normal lung tissues (n = 328). Violin plots show log10(RPM.filtered + 1) for the American HDM chromosome and scaffold assemblies and the European HDM scaffold and contig assemblies. Gray lines connect matched tumor-normal pairs. Two-sided paired Wilcoxon p-values are shown for each assembly. **B)** Comparison of candidate HDM-aligned read levels across adjacent normal lung tissue, smoker, and never-smoker tumor groups for each HDM reference assembly. Violin plots show log10(RPM.filtered + 1), with individual samples overlaid and red dots indicating the median. Kruskal-Wallis p-values, indicating overall group differences, and pairwise Wilcoxon rank-sum p-values adjusted using the Benjamini-Hochberg method are shown above the plots. Statistical analyses were performed on raw RPM values, whereas log10-transformed values are displayed for visualization.

Following filtering, we quantified reads aligned to HDM reference assemblies as RPM (**Supplementary Table 5**) and compared RPM values between tumors and matched normal samples. Among the 328 subjects with matched adjacent normal lung tissue, paired-end reads aligned to the American HDM scaffold were modestly enriched in tumors relative to matched tissue normals (paired Wilcoxon p = 7.88 × 10^−4^), whereas no significant difference was observed for the American HDM chromosome assembly (p = 0.169) (Fig. 3A). For European HDM, the contig assembly showed a modest increase in tumors compared with tissue normals (p = 0.0318), whereas the scaffold assembly showed no significant difference (p = 0.57).

Singleton-read analyses were performed as a secondary assessment because singleton reads lack mate-pair support and therefore provide lower mapping confidence than paired-end reads. Nevertheless, singleton analyses largely recapitulated the paired-end findings, showing similar tumor-normal trends across most HDM reference assemblies, although not all comparisons reached significance. Therefore, singleton results were considered supportive rather than primary evidence (**Supplementary Fig. 3**).

HDM-aligned RPM values differed significantly across tissue normal, smoker, and never-smoker groups for both American and European HDM assemblies (Fig. 3C). For American HDM, both chromosome and scaffold assemblies showed significant group differences (Kruskal-Wallis p = 9.3 × 10^−46^ and p = 2.3 × 10^−29^, respectively). Smokers consistently exhibited higher HDM-aligned RPM values than never-smokers across both American assemblies, and similar patterns were observed for European HDM scaffold and contig assemblies (all pairwise p < 0.001).

Collectively, these analyses demonstrate that stringent alignment filtering identifies reproducible HDM-aligned signals across multiple reference assemblies while revealing consistent differences according to tissue source and smoking status. However, because alignment-based approaches alone cannot exclude cross-mapping to related organisms or conserved genomic regions, these reads were considered HDM-associated rather than definitively HDM-derived. Therefore, orthogonal taxonomic validation using BLAST-based annotation was performed in subsequent analyses.

### BLAST-supported HDM detection is sparse and assembly-dependent across tumor and adjacent normal samples

Because available HDM reference genomes vary in completeness and sequence contiguity, and alignment alone cannot exclude cross-mapping to related organisms, candidate HDM-aligned reads were further validated using BLAST against the NCBI nr/nt database (see Methods). Given the unknown abundance of HDM-derived DNA in human lung tumors and the potential effects of DNA degradation, host clearance, exposure timing, and the overwhelming excess of human DNA, we adopted a conservative exploratory strategy for HDM-supported sample classification.

**Figure 4:**
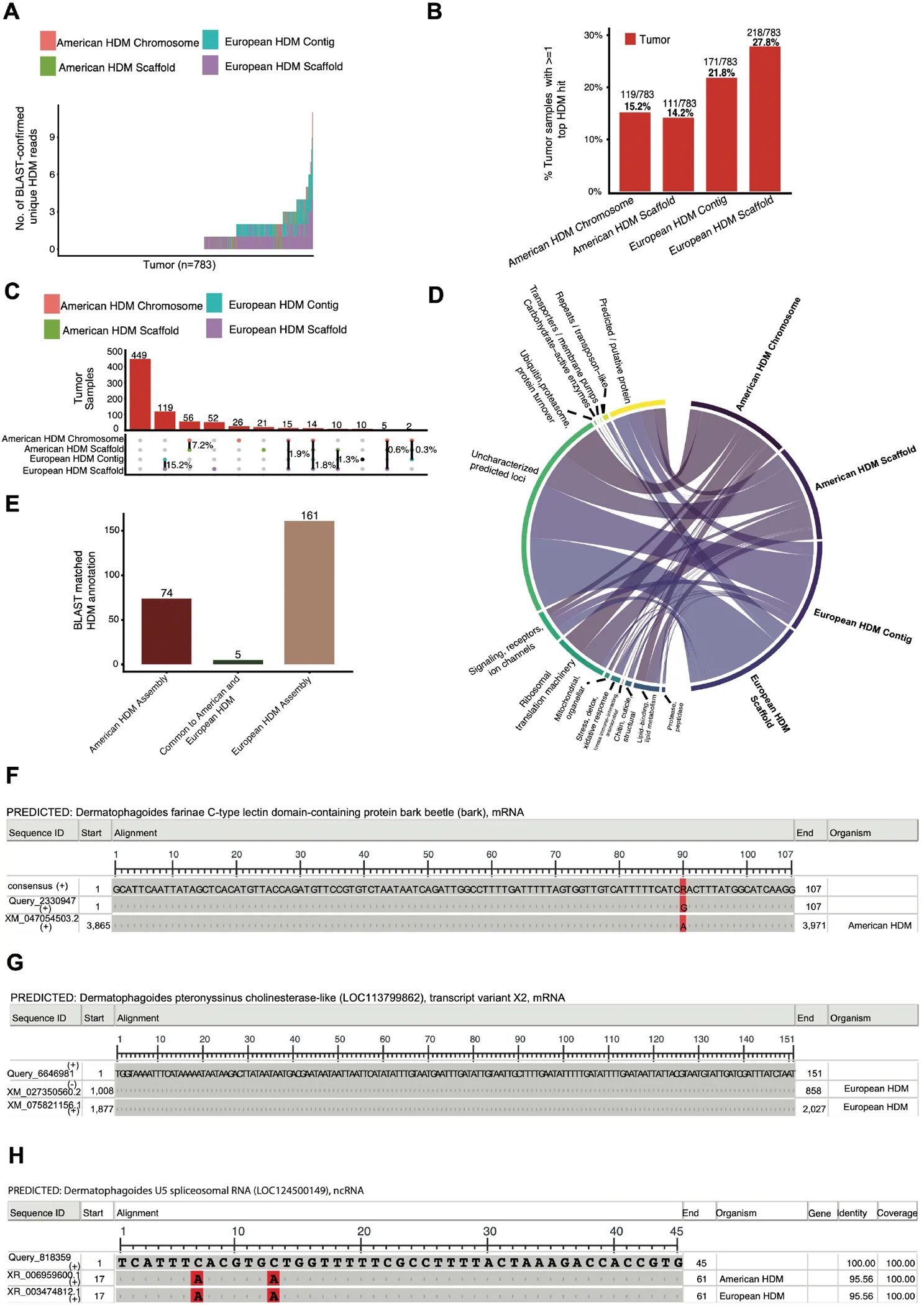
BLAST-supported HDM detection across tumor samples and HDM reference assemblies. A) Distribution of BLAST-supported unique HDM read counts across tumor samples (n = 783). Tumor samples are ordered by the number of BLAST-supported unique reads (x-axis), and the y-axis indicates the number of unique reads per sample. Colors denote the HDM reference assembly for which each read was assigned a top BLAST hit, including American HDM chromosome, American HDM scaffold, European HDM contig, and European HDM scaffold assemblies. B) Percentage of tumor samples classified as HDM-detected for each HDM reference assembly. HDM detection was defined as the presence of at least one unique read whose BLAST hit matched the corresponding HDM reference. C) UpSet plot showing the overlap of HDM-detected tumor samples across the four HDM reference assemblies. Bars indicate the number of tumors detected by individual assemblies or assembly combinations, whereas connected dots indicate the corresponding overlap patterns. D) Chord diagram showing the relationship between HDM reference assemblies and the functional annotations of their top BLAST hits. Links connect each HDM assembly to the corresponding annotated HDM gene or sequence category identified by BLAST. E) Classification of BLAST-supported HDM annotations as American HDM-specific, European HDM-specific, or shared between both HDM species. Shared annotations represent top BLAST hits identified using both American and European HDM reference assemblies. F-H) Representative BLAST alignments illustrating HDM-supported reads mapping to annotated HDM sequences. Examples include an American HDM-specific alignment (F), a European HDM-specific alignment (G), and an alignment shared between both HDM reference species (H). These examples are provided for illustration and demonstrate the alignment patterns supporting BLAST-based HDM classification.

BLASTN was performed on all HDM-aligned reads (MAPQ ≥ 30; matched alignment length ≥ 40 bp) from paired-end and singleton datasets across all 783 tumor-normal pairs. Because a single query read can produce multiple database matches, each read was evaluated using only its highest-confidence BLAST alignment. Reads were considered HDM-supported only when the highest scoring hit, or a tied highest-scoring hit, matched the corresponding *Dermatophagoides* species represented by the reference assembly. Each query read was counted once, regardless of the number of BLAST alignments, thereby avoiding inflation of read counts due to multiple database hits for the same read. Using this conservative approach, BLAST-supported HDM read counts remained low, ranging from 0 to 10 unique reads per sample across all reference assembly (Fig. 4A and **Supplementary Table 6**). Thus, samples containing at least one BLAST-supported unique read were classified as HDM-supported for exploratory downstream analyses rather than as a quantitative measure of HDM burden (**Supplementary Table 7**). Each unique read was counted once, even when the same read produced multiple HDM alignments.

The proportion of HDM-supported tumors varied by reference assembly. European HDM assemblies identified the highest proportion of supported tumors, with 171 of 783 tumors (21.8%) supported by the contig assembly and 218 of 783 tumors (27.8%) supported by the scaffold assembly. In comparison, American HDM assemblies identified 119 of 783 tumors (15.2%) using the chromosome assembly and 111 of 783 tumors (14.2%) using the scaffold assembly (Fig. 4B). The higher detection rate observed for European HDM assemblies may reflect differences in sequence representation, assembly fragmentation, or species-specific sequence similarity. A direct comparison would require a chromosome-level European HDM reference genome.

We next evaluated the concordance of HDM-supported calls across reference assemblies (Fig. 4C). Most tumors (449/783, 57.3%) lacked BLAST-supported HDM reads, and overlap between assemblies was modest. Among tumors, 119 (15.2%) were supported by both European HDM assemblies, whereas 56 (7.2%) were supported by both American HDM assemblies. Because overlap was assessed at the sample level, concordant classification does not necessarily imply that identical reads or BLAST alignments were shared between assemblies.

To characterize the biological origin of BLAST-supported reads, top-hit annotations were grouped into 14 functional categories based on dominant annotation keywords (Table 2). Most HDM-supported reads mapped to uncharacterized or predicted loci, ribosomal and translation-associated genes, and mitochondrial or other organellar genes (Fig. 4D and **Supplementary Table 8**). Smaller numbers of reads mapped to proteases and peptidases, lipid metabolism-related proteins, and chitin- or cuticle-associated structural components.

Comparison of BLAST-supported annotations across HDM species showed that European HDM assemblies yielded more unique annotations (161) than American HDM assemblies (74), whereas only five annotations were shared between the two species (Fig. 4E and **Supplementary Table 9**). Representative BLAST alignments illustrating American HDM-specific, European HDM-specific, and shared annotations are shown in Fig. 4F–H. In each case, the retained reads showed their strongest BLAST support for annotated *Dermatophagoides* sequences under the conservative read-counting strategy used in this study.

### Molecular alterations associated with HDM detection in tumors

**Figure 5:**
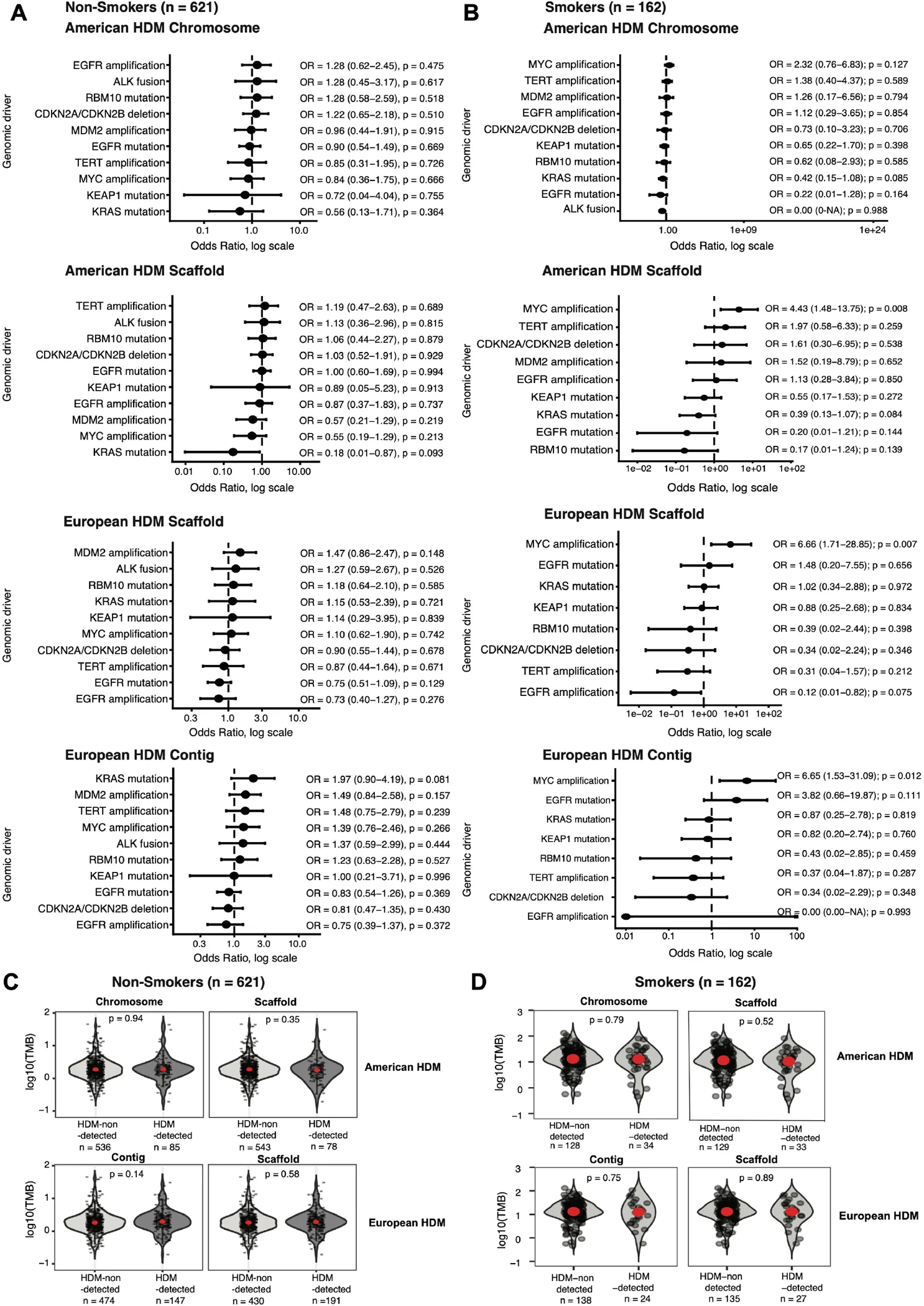
Association between HDM detection, recurrent genomic alterations, and TMB by smoking status. A-B) Multivariable logistic regression analyses evaluating associations between recurrent genomic alterations and HDM detection in never-smokers (A, n = 621) and smokers (B, n = 162). Separate models were fitted for each HDM reference assembly (American HDM chromosome and scaffold; European HDM scaffold and contig). HDM detection status (HDM detected vs. HDM-non-detected) was modeled as the binary outcome, with recurrent genomic alterations (*ALK* fusion; *CDKN2A/CDKN2B* deletion; *EGFR, KEAP1, KRAS,* and *RBM10* mutations; *EGFR, MDM2, MYC,* and *TERT* amplifications) included as predictor variables. Forest plots show adjusted odds ratios (ORs) with 95% confidence intervals (CIs) on a logarithmic scale. The vertical dashed line indicates OR = 1, and p-values correspond to two-sided Wald tests for individual regression coefficients. C-D) Comparison of TMB between HDM-non-detected and HDM-detected samples in never-smokers (C) and smokers (D) across American and European HDM reference assemblies. Violin plots show the distribution of log10-transformed TMB values, with individual tumors overlaid and red dots indicating the median. Statistical significance was assessed using a two-sided Wilcoxon rank-sum (Mann-Whitney U) test. Multiple-testing correction was performed using the Benjamini-Hochberg FDR method, although the p-values displayed in the figure are nominal (uncorrected) and correspond to comparisons between HDM-detected and HDM-non-detected tumors for each HDM reference assembly.

Based on previous reports that HDM can induce DNA damage^14,15^, we examined whether HDM detection was associated with recurrent lung cancer driver alterations, including *EGFR*, *KRAS*, *RBM10*, and *KEAP1* mutations, *ALK* fusion, *MDM2*, *MYC*, and *TERT* amplifications, and *CDKN2A/CDKN2B* deletion in never-smokers (n = 621/783 tumors) (**Supplementary Table 10**). Stratified logistic regression identified no significant association between HDM detection and the evaluated genomic drivers in never-smokers across the American HDM chromosome/scaffold and European HDM scaffold/contig assemblies (Fig. 5A). In smokers, *MYC* amplification showed nominal associations with higher odds of HDM detection across the American HDM scaffold (odds ratio [OR] = 4.43, p = 0.008), European HDM scaffold (OR = 6.68, p = 0.007), and European HDM contig (OR = 6.65, p = 0.012) assemblies (Fig. 5B). However, these associations did not remain significant after correction for multiple testing. No consistent associations were observed for the remaining genomic alterations, and TMB did not differ significantly between HDM-detected and non-detected tumors in either never-smokers or smokers across all evaluated assemblies (Fig. 5C–D). Overall, these findings do not support an association between HDM detection and a mutagenic effect on the lung genome (**Supplementary Table 11**).

### HDM detection is associated with immune and microbiome features in tumors

Building on our previous experimental studies suggesting that HDM exposure promotes lung tumorigenesis through immune-mediated rather than mutagenic mechanisms^16,17^, we next analyzed immune cell infiltration in tumors with available bulk RNA-seq data (n = 627/783, **Supplementary Table 12**). Immune cell composition was inferred using the xCell algorithm^25,26^ and compared between HDM-detected and HDM-non-detected tumors across HDM reference assemblies.

**Figure 6:**
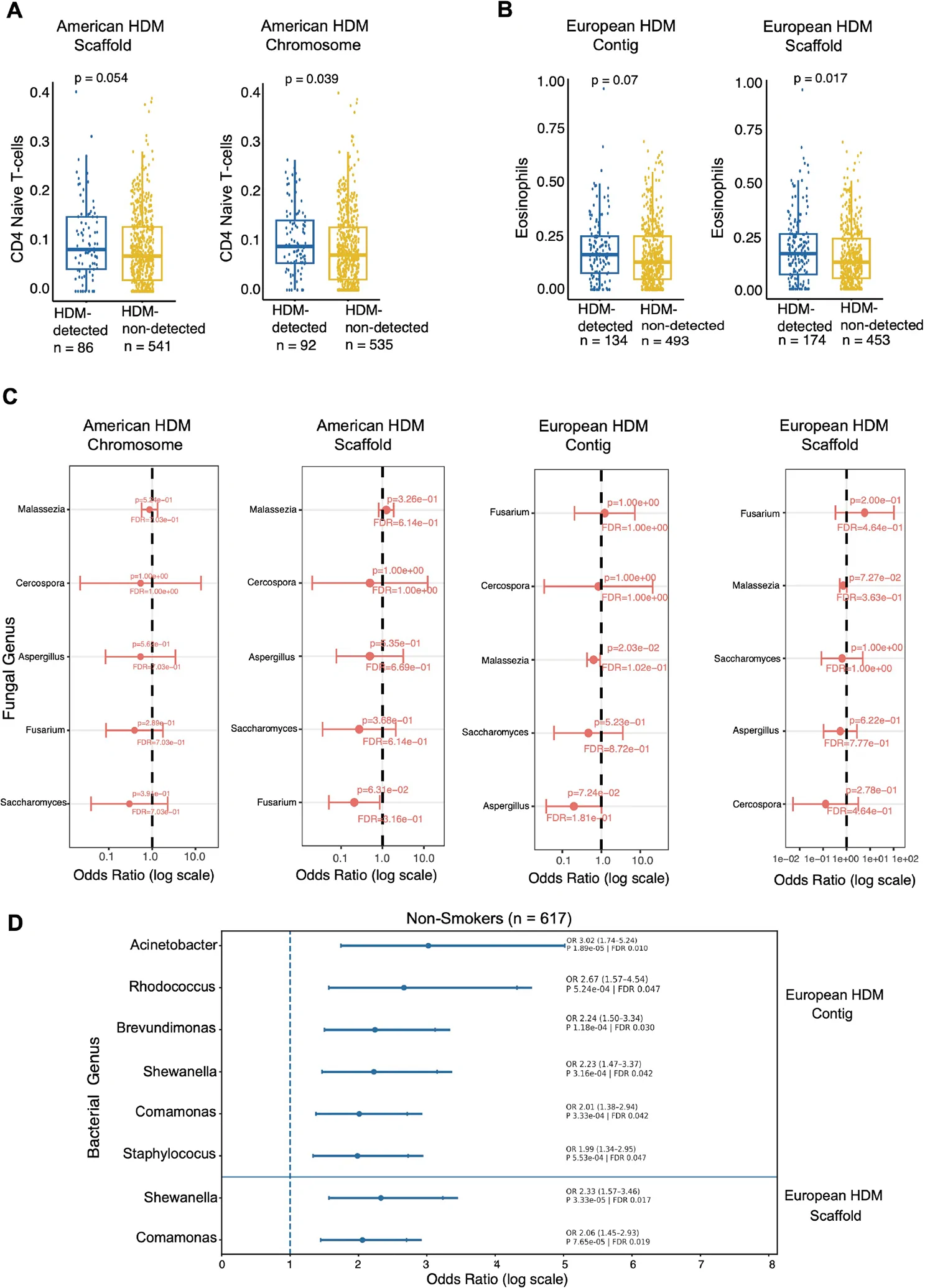
Immune and microbial associations with HDM detection. A-B) Comparison of CD4 naïve T-cell (A) and eosinophil (B) abundance between HDM-detected and HDM-non-detected tumors across the American HDM scaffold and chromosome assemblies (A) and the European HDM contig and scaffold assemblies (B), respectively, among tumors with available RNA-seq data (n = 627). Boxplots show individual samples overlaid. Nominal p-values are shown; statistical significance was assessed after Benjamini-Hochberg FDR correction. C-D) Associations between HDM detection and fungal (C) and bacterial (D) genera in never-smoker tumors (n = 617). Forest plots display odds ratios with 95% confidence intervals, together with nominal p-values and FDR-adjusted values.

Among never-smokers, American HDM detection was associated with higher naïve CD4^+^ T-cell abundance for both the scaffold (p = 0.054) and chromosome (p = 0.039) assemblies (Fig. 6A), although these associations did not remain significant after false discovery rate (FDR) correction. European HDM detection was associated with increased eosinophil abundance, reaching nominal significance for the scaffold assembly (p = 0.017) and showing a similar trend for the contig assembly (p = 0.07) (Fig. 6B). Although these associations were not significant after multiple-testing correction, the directionally consistent trends across assemblies suggest modest immune changes associated with HDM detection.

Among smokers, RNA-seq data were available for only 88 of 162 tumors, with relatively few HDM-detected cases across all reference assemblies (12–22 samples depending on the assembly). No significant differences in immune cell composition were observed in this subgroup, likely reflecting the limited statistical power (**Supplementary Table 13**).

Finally, we examined whether HDM detection was associated with the tumor microbiome. Because bacteria and fungi can inhabit HDM or form part of its complex microbiome^27^ and because prior studies have reported associations between the lung microbiome and lung cancer^28,29^, we analyzed contamination-filtered microbiome abundance estimates from a recent Sherlock-Lung study^21^. Microbiome analyses were restricted to 617 of 621 never-smoker tumors, as EAGLE smokers were not included in the original microbiome study and four Sherlock-Lung tumors were excluded during contaminant filtering.

Genus-level differential abundance analysis was performed for 495 bacterial and 5 fungal genera by comparing HDM-detected and HDM-non-detected tumors. No fungal genera were significantly associated with HDM detection after multiple-testing correction (Fig. 6C and **Supplementary Table 14**). In contrast, the European HDM contig analysis identified six bacterial genera with FDR-significant differential abundance (*Acinetobacter, Rhodococcus, Brevundimonas, Shewanella, Comamonas*, and *Staphylococcus*), whereas the European HDM scaffold analysis identified two (*Shewanella* and *Comamonas*) (Fig. 6D and **Supplementary Table 15**). Notably, *Shewanella* and *Comamonas* were consistently identified across both European HDM analyses, supporting the reproducibility of these bacterial associations.

Overall, these findings suggest that HDM-derived DNA may be detectable in tumors from both smoker and never-smoker patients and may be associated with differences in the tumor immune microenvironment and microbiome composition.

## Discussion

To our knowledge, this is the first study to report the detection of HDM-derived DNA in human lung tumors. Using stringent alignment filtering, multiple HDM reference assemblies, and BLAST-based taxonomic validation, we identified low-abundance HDM-supported DNA in a subset of tumors from both never-smokers and smokers. Although HDM detection was not significantly associated with TMB or recurrent genomic alterations in either smokers or never-smokers, it was associated with modest differences in immune cell composition across most American and European HDM reference assemblies and with a limited but reproducible bacterial co-detection pattern in never-smokers identified using the European HDM assemblies. Together, these findings establish a comparative genomic framework for detecting HDM-derived DNA in human tumors and indicate possible species- or assembly-specific associations, suggesting that HDM exposure may influence the lung tumor microenvironment through immune- and microbiome-related mechanisms rather than direct mutagenesis. Additional studies are needed to determine whether long-term exposure to common aeroallergens such as HDM plays a causal role in lung tumor initiation or progression.

Microbial DNA has been detected in multiple cancer types, although the detection and interpretation of low-biomass microbial signals remain subjects of ongoing debate because of the potential for environmental contamination, reagent-derived nucleic acids, and analytical artifacts^30,31,32,33^. HDM represents one of the most common indoor aeroallergens and environmental exposures worldwide, linked to chronic airway inflammation and epithelial barrier dysfunction^11,12,13^. Experimental studies have further shown that HDM exposure induces oxidative stress and DNA double-strand breaks in airway epithelial cells^14,15^, raising the possibility that HDM could contribute to lung tumorigenesis through mutagenic mechanisms. In parallel, our previous studies demonstrated that chronic HDM exposure accelerates lung tumor progression in multiple mouse models by promoting persistent inflammation followed by immune remodeling of the tumor microenvironment^16,17^. However, whether HDM DNA remnants are detectable in human lung tumors and whether chronic HDM exposure contributes to lung carcinogenesis in humans have remained unknown.

To address these questions, we analyzed unmapped non-human reads using multiple reference assemblies representing the two predominant HDM species, *Dermatophagoides farinae* and *Dermatophagoides pteronyssinus*. Because currently available HDM genomes remain incomplete and differ substantially in assembly quality, we compared chromosome-, scaffold-, and contig-level references and incorporated BLAST-based validation to minimize false-positive assignments. Although HDM detection varied across reference assemblies, the overall pattern was consistent, supporting the robustness of the detection strategy while underscoring the importance of high-quality reference genomes for future environmental metagenomic studies.

Functionally, HDM-supported reads mapped to gene categories including proteases/peptidases and chitin- or cuticle-associated structural components, consistent with known biological properties of HDM, including allergenicity, epithelial barrier disruption, and environmental persistence. Although these annotations represented only a minority of the supported reads relative to housekeeping genes, their reproducible detection across reference assemblies supports the presence of diverse HDM-derived sequences rather than enrichment of a single functional category.

Despite identifying HDM-aligned DNA in a subset of tumors, most positive samples contained only a few BLAST-supported reads, consistent with the low microbial biomass previously reported in Sherlock-Lung tumors^21^. Importantly, HDM detection was not associated with recurrent driver alterations or TMB, arguing against a major mutagenic contribution of chronic HDM exposure, at least when HDM-derived DNA detected in lung tumors is used as a surrogate marker of exposure. Instead, HDM detection was accompanied by modest alterations in immune cell composition, including increased naïve CD4^+^ T-cell and eosinophil infiltration, together with limited but reproducible bacterial co-detection. These observations are consistent with our previous experimental studies, in which HDM promoted lung tumor progression primarily through chronic inflammation and alterations of the tumor immune microenvironment rather than direct mutagenic effects^17^.

Our findings are consistent with the broader concept of non-genotoxic carcinogenesis. Unlike classical genotoxic carcinogens, non-genotoxic agents do not directly damage DNA but instead promote tumor development through mechanisms such as chronic inflammation, oxidative stress, epigenetic reprogramming, immune dysregulation, and altered cellular signaling pathways^34,35,36^. Notably, a substantial proportion of established human carcinogens lack detectable mutagenic activity^35^. In this context, the absence of consistent associations between HDM detection and recurrent driver alterations or TMB, together with the modest immune changes observed in HDM-detected tumors, is consistent with the possibility that HDM exposure may contribute to lung tumor development through indirect, non-genotoxic mechanisms.

Among the immune changes identified, eosinophil enrichment is particularly intriguing because it may represent a potential immunological signature of allergen-associated tumor microenvironments. Eosinophils are classically associated with allergic inflammation and host defense against parasites, although their role in cancer is complex and context dependent. In lung cancer, tumor-associated tissue eosinophilia has frequently been associated with favorable prognosis and improved responses to immunotherapy, potentially through vascular normalization and increased CD8⁺ T-cell infiltration^37,38^. Conversely, eosinophils may also promote tumor progression through extracellular matrix remodeling, angiogenesis, or immunosuppressive polarization^38,39^. Likewise, increased naïve CD4⁺ T-cell infiltration has been associated with a more tumor-permissive immune state in LUAD^40^. Although neither immune association remained significant after multiple-testing correction, the consistent direction of these changes across HDM reference assemblies supports further investigation into whether chronic aeroallergen exposure contributes to remodeling of the lung tumor immune microenvironment.

We also identified modest associations between HDM DNA detection and the tumor microbiome in never-smokers. HDM harbors a complex microbiome that reflects the broader indoor dust environment^41,42^, providing a biological plausibility for the bacteria genera identified in HDM-detected tumors. Although these findings do not establish a direct relationship between HDM and individual bacterial taxa, previous studies have consistently reported *Acinetobacter* and *Staphylococcus* within HDM-associated microbial communities^41,42^, while both genera have also been described in some lung cancer microbiome studies^43^. The reproducible association of *Shewanella* and *Comamonas* across independent HDM reference assemblies further supports the robustness of the observed bacterial co-detection pattern. Together, these findings raise the possibility that chronic exposure to indoor dust-associated microbial communities contributes to the composition of the lung tumor microbiome.

Tumors consistently exhibited modestly higher HDM-derived DNA than adjacent normal tissues across multiple reference assemblies. Although these differences were small, they suggest that HDM-derived DNA may be retained or enriched within the tumor microenvironment. HDM-derived DNA was also detected at higher levels in smokers than in never-smokers. Although the underlying explanation remains unclear, smoking-induced airway injury, chronic inflammation, impaired mucociliary clearance, or increased epithelial permeability could plausibly facilitate the persistence or detection of exogenous DNA within the respiratory tract. Whether these observations reflect a biological role for HDM in lung tumorigenesis or, alternatively, differences in the retention of extracellular environmental DNA remains unknown and warrants further investigation.

Several limitations should be considered when interpreting these findings. HDM-derived DNA was detected at very low abundance, with most positive tumors containing only a few BLAST-supported reads. Consequently, the study was likely underpowered to detect subtle clinical or molecular associations, and the true magnitude of HDM exposure within tumors remains uncertain. In addition, currently available HDM reference genomes remain incomplete and are predominantly limited to draft scaffold- and contig-level assemblies. Although our multi-assembly strategy and BLAST-based validation improved confidence in taxonomic assignment, incomplete reference genomes likely reduced detection sensitivity and may have underestimated the true prevalence of HDM-derived DNA. Future chromosome-scale assemblies, together with long-read sequencing approaches, should substantially improve detection accuracy. Furthermore, BLAST confirmation provides read-level evidence supporting HDM detection but does not directly quantify HDM biomass. Because short sequencing reads may share sequence similarity with related organisms or conserved genomic regions, taxonomic assignment remains probabilistic rather than definitive. Orthogonal validation using targeted molecular assays, such as quantitative PCR, digital PCR, or targeted capture sequencing, will be important to independently confirm the presence and abundance of HDM-derived DNA in human lung tumors. The relatively small smoker cohort also limited statistical power for subgroup analyses. Future studies should include larger smoker cohorts, evaluate additional histological subtypes beyond LUAD, and determine whether HDM-derived DNA is similarly detectable in LUSC and small cell lung cancer (SCLC), which are more prevalent among smokers. Finally, the present study focused exclusively on HDM. Future investigations should determine whether DNA derived from other common aeroallergens, including pollens and animal dander, which have also been reported to induce oxidative stress and DNA damage^44^, is detectable in human lung tumors. It will also be important to determine whether chronic exposure to these and other common aeroallergens contributes to lung carcinogenesis through immune-mediated or other nongenotoxic mechanisms, and under specific conditions, whether such exposures may also promote mutagenic processes. Elucidating these mechanisms will help determine whether these exposures represent previously underrecognized environmental risk factors for lung cancer.

## Supplementary Material

Supplementary Files

This is a list of supplementary files associated with this preprint. Click to download.

• SupplementaryTables.xlsx

• SupplementaryFigures.docx

## Figures and Tables

**Table 1:** HDM genome assemblies used in this study

HDM Species	Assembly Level	GenBank Accession	Annotation	Release date	Scaffolds	Genome Size (Mb)	Total ungapped length (Mb)	Genome Coverage (x)	Scaffold N50	No. of chromoso
*D. Farinae*	Chromosome	GCA_024713945.1	NCBI RefSeq	Aug 2022	21	62.4 Mb	62.4	96	7.1 Mb	10
	Scaffold	GCA_000767015.2	Submitter	Feb 2022	10	60.4 Mb	60.1	431	9 Mb	NA
*D. Pteronyssinus*	Scaffold	GCA_027571235.1	NCBI RefSeq	Jan 2023	107	56.7 Mb	56.7	309	2.7 Mb	NA
	Contig	GCA_003076615.3	Submitter	Feb 2022	131	59 Mb	59	170	963.1 kb	NA

**Table 2:** Functional classification of BLAST-supported HDM reads based on keyword matching of subject sequence annotations

Functional class	Keyword criteria in BLAST subject annotation (stitle)
**Mitochondrial / organellar**	mitochondrial, COX, cytochrome, ND, ATP synthase, 12S, 16S, tRNA
**Ribosomal / translation machinery**	rRNA, ribosomal, elongation factor, initiation factor, tRNA synthetase/ligase, polymerase, spliceosome
**Proteases / peptidases**	protease, peptidase, serine protease, cysteine protease, metalloprotease, trypsin, cathepsin
**Lipid-binding / membrane-related**	lipid, lipase, phospholipase, fatty acid, apolipoprotein, binding protein, transporter
**Cuticle / chitin / structural**	chitin, cuticle, collagen, keratin-like, actin, tubulin, myosin
**Stress / detox / oxidative response**	heat shock protein (HSP), oxidoreductase, dehydrogenase, peroxidase, superoxide, glutathione S-transferase (GST)
**Signaling / receptors / ion channels**	kinase, phosphatase, GTPase, receptor, GPCR, ion channel, calcium, potassium
**Uncharacterized / predicted loci**	uncharacterized, LOC

## Data Availability

WGS data analyzed in this study are available through the dbGaP database under accession numbers phs001697.v2.p1 (Sherlock-Lung study) and phs002992.v1.p1 (EAGLE study). Normal and tumor-paired CRAM files for WGS subjects from the Sherlock-Lung and EAGLE studies have been deposited under these accession numbers, respectively. RNA-seq data from the Sherlock-Lung study analyzed in this work are available through dbGaP under accession number phs003955.v1.p1. The analysis code used in this study is publicly available at https://github.com/sharmas30-94/House_Dust_Mite. Requests for further information and resources should be directed to and will be fulfilled by the lead contacts, Dr. Maria Teresa Landi (landim@mail.nih.gov) and Dr. Samuel Bertin (sbertin@ucsd.edu).
